# Clinical performance of two chemomechanical caries removal agents in primary molars: a randomized controlled trial

**DOI:** 10.1007/s40368-025-01030-9

**Published:** 2025-04-09

**Authors:** M. Maashi, H. Elkhodary, N. Bamashmous, O. Felemban, N. Alamoudi

**Affiliations:** 1 Ministry of Health, Jeddah, Saudi Arabia; 2 Pediatric Dentistry Department, Faculty of Dentistry, King Abdulaziz University, P.O. Box 80200, 21589 Jeddah, Saudi Arabia; 3 Department of Pedodontics and Oral Health, Faculty of Dental Medicine for Girls, Al Azhar University, Cairo, Egypt

**Keywords:** BRIX3000, Carie-care, Chemomechanical caries removal, Caries removal, Pain, Primary molars

## Abstract

**Purpose:**

The concept of Chemomechanical Caries Removal (CMCR) includes the selective removal of caries-infected tissue while preserving caries-affected tissue. However, studies examining its application to children are scarce. The aim was to assess pain perception and time duration of CMCR agents when removing caries in primary molars in a sample of children compared to the conventional method of caries removal using rotary burs.

**Methods:**

A randomized controlled clinical trial with a split-mouth design with a sample of 60 children aged 4–9 years with 120 cavitated occlusal carious primary molars was equally distributed into 2 experiments: BRIX3000^®^ vs. conventional method (Experiment 1) and Carie-Care™ vs. conventional method (Experiment 2). The time duration of caries removal was recorded. Perception of pain during caries excavation was evaluated using the “Wong-Baker FACES^®^ Pain Rating Scale.”

**Results:**

Caries removal using the conventional method was significantly faster compared to CMCR agents in both experiments (*P < *0.001 and *P < *0.001). Caries removal using BRIX3000^®^ was significantly faster than Carie-Care™ (*P < *0.001). Caries removal with BRIX3000^®^ or Carie-Care™ were reported less painful than the conventional method (*P* = 0.002 and *P* = 0.011, respectively).

**Conclusion:**

The study concluded that although CMCR methods require more time for caries removal, they were reported to be less painful.

**Trial registration:**

The study protocol was registered at www.clinicaltrials.gov under the identifier NCT05427591.

## Introduction

In their search for an effective way to treat and manage dental caries, dentists have long viewed it as a major challenge (Selwitz et al. 2007). The conventional method for treating dental caries involves the use of burs attached to handpieces and hand excavator instruments to remove the carious tissues (Jawa et al. 2010). The chemomechanical caries removal (CMCR) technique is built on the principles of minimal invasive dentistry (MID). The CMCR method requires the chemical dissolution of the decayed dentin, which will be removed gently with hand instruments (Hamama et al. 2014). It differs from the conventional procedure in that it selectively removes carious dentin, so it is less destructive and less painful (Goomer et al. 2013; Soni et al. 2015). The CMCR technique has been found to reduce the need for local anesthesia (LA) and lower the possibility of accidental pulp exposures. As a result, it is more accepted and less stressful, particularly for individuals who are severely apprehensive, handicapped, and children (Hamama et al. 2014). Multiple CMCR agents have been introduced into the market since 1975. These agents were mostly manufactured as sodium hypochlorite (NaOCl)-based. However, there has been a movement towards manufacturing these agents primarily as enzyme-based agents which has antibacterial and anti-inflammatory effects (Felizardo et al. 2018; Hamama et al. 2014).

The two most recent enzyme-based CMCR agents are Carie-Care™ and BRIX3000^®^. Carie-Care™ offers specific benefits compared to current CMCR agents. It is distinct from older enzyme-based CMCR agents since it does not contain NaOCl or any other potent chlorinating agents (Rajakumar et al. 2013). Carie-Care™ contains natural components such as Clove oil, which possesses anti-inflammatory, mild anesthetic, analgesic, antibacterial, and antioxidant characteristics (Nisar et al. 2021). The newest CMCR agent named BRIX3000^®^ by the laboratory Brix Medical Science has been released recently in the dental market in 2012. In addition to the advantages of CMCR agents, this agent holds clinical significance as it is manufactured with a unique technology known as Encapsulating Buffer Emulsion (EBE) (Felizardo et al. 2018; Prabhav et al. 2019). This technology, according to the manufacturer, provides the product with distinctive properties that increase the effectiveness and efficiency of caries removal, resulting in reduced time needed for dental treatment (Ismail and Al Haidar 2019; Torresi and Besereni 2017).

Upon reviewing the existing literature, it has been observed that the use of CMCR agents in pediatric patients is relatively insufficient. Furthermore, there is a scarcity of studies that have compared the convenience of CMCR agents with conventional methods among children using a split-mouth randomized controlled design. The objective of the present study is to clinically evaluate caries removal time and pain experience of two CMCR agents, namely BRIX3000^®^ and Carie-Care™, in comparison with conventional methods for treating primary molars in children. The null hypothesis of the study claimed that there is no difference in pain experience or working time when excavating caries in primary molars between the CMCR agents (BRIX3000® and Carie-Care^TM^) and the conventional approach.

## Materials and methods

The study was a randomized controlled clinical trial with a split-mouth design that included 2 experiments. In one experiment, Carie-Care™ was compared to the conventional technique, while in the other experiment, BRIX3000^®^ was compared to the conventional technique. Approval for conducting the study according to the guidelines of the Declaration of Helsinki was obtained from the Research Ethics Committee at the Faculty of Dentistry King Abdulaziz University, under approval number 107-06-19. The research protocol was registered at Clinicaltrials.gov under the identifier NCT05427591. The reporting of the study follows the Consolidated Standards of Reporting Trials (CONSORT) guidelines for reporting randomized trials (Moher et al. 2012).

Children who sought dental treatment at the Pediatric Dental Clinics at King Abdulaziz University Dental Hospital during the period from October 2022 to December 2023 were screened for eligibility to participate in the study. Inclusion criteria encompassed the following: being healthy with an ASA 1 classification, aged 4–9 years, cooperative behavior for dental treatment according to the “Frankl Behavioral Rating Scale” (rated as “definitely positive” or “positive”), and no known allergies to any dental materials used in the study. In addition, the subjects had to have at least two primary molars on each side with cavitated occlusal caries of moderate depth, similar width, softened brown dentin, and absence of pulpal necrosis symptoms. Maxillary and mandibular primary molars (1st or 2nd) were included. Exclusion criteria comprised primary molars associated with moderate to severe periodontal diseases, mobile or restored molars, non-vital molars, non-restorable molars, molars with arrested caries or developmental defects, periapical or furcal radiolucency on radiographs, and primary molars with more than half of the root length resorbed. Informed written consents were signed by the parents after a detailed explanation was given about the procedure of the treatment along with its possible outcomes, risks, benefits, and discomforts.

### Sample size determination

Based on the results of Kumar et al. (2016), when the number of pairs is 27 in each experiment, a paired group design will have 80.67% power to reject the null hypotheses, assuming that the expected mean of the paired differences in the mean time is 7.5 min, the standard deviation of the paired differences is 3 min and that each test is made at the 5% level. When the number of pairs is 26 in each experiment, a signed-rank Wilcoxon test of the null hypotheses of no Wong Baker Pain Rating Scale median difference will have 81.54% power to detect a difference in medians between groups at the 5% two-sided level. A sample size of 30 pairs of teeth in each experiment will have 80% power to detect a probability of 30% that an observation in the CMCR Group is less than an observation in the conventional Group using a Mann–Whitney test with a 0.05 two-sided significance level.

### Study groups

The included teeth were assigned randomly using envelopes into either experiment 1 or experiment 2 according to caries removal methods. Experiment 1 included 30 children with 60 primary molars who underwent caries removal by the CMCR agent BRIX3000^®^ (the experimental group) on one molar (*n = *30) and the conventional method (the control group) on the contralateral molar (*n = *30). Experiment 2 encompassed 30 children with 60 primary molars who underwent caries removal by the CMCR agent Carie-Care™ (the experimental group) on one molar (*n = *30) and the conventional method (the control group) on the contralateral molar (*n = *30).

### Randomization

For the randomization process, the intervention assignments were enclosed in sixty identical envelopes. Experiment 1 utilized a total of thirty envelopes, with half of them (fifteen) containing instructions to do BRIX3000^®^ before the conventional procedure, and the other half containing instructions for the reverse sequence. For the 30 envelopes utilized in experiment 2, half of them (15 envelopes) directed the operator to do Carie-Care™ first followed by the conventional method, whereas the remaining 15 envelopes instructed the opposite order. All 60 envelopes were mixed and placed into a container. Each participant selected an envelope. To control the side as a variable (right vs. left), it was decided that the first intervention, according to the instructions in the envelope, would be conducted on the patient's right side and followed by the second intervention on the left side*.*

### Clinical procedures

The clinical procedures of the study were performed by a pediatric dentist (M.M.). Each tooth was treated in a separate session with a minimum gap of one week. A periapical radiograph was taken preoperatively to reveal the extent of caries advancement and ruling out periapical or furcal radiolucency. Prior to the commencement of the dental procedures in all groups, topical anesthetic (Benzocaine 20%) was applied then local anesthetic (Scandicaine 2%, Mepivacaine hydrochloride 0.020 g/mL, and adrenaline base 0.010 mg/mL) agents were administered. Then, teeth were isolated using a rubber dam. The level of dental caries was clinically assessed using a dental probe. Caries removal was done utilizing one of the 3 methods as follows:

#### Experiment 1 (caries removal utilizing Brix3000^®^)

BRIX3000^®^ was administered to the cavity as manufacturer’s instructions with double-ended, blunt-edged dental spoon excavator based on the cavity size (Size #2, #3, #4; Nordent Manufacturing Inc., Bonnie Lane, Elk Grove Village, IL, USA) leaving it to act for 2 min. Oxygen bubbles were seen throughout that time, and the gel’s original translucent green appearance had changed to one that was hazy. A moistened cotton pellet was used to remove the gel. A dry, sterile cotton pellet and a gentle air blow were used to dry the cavity after it had been washed with water. Then, the infected tissues were removed through curettage by a dental excavator utilizing pendulum movement with no pressure applied, first at the surrounding walls followed by the pulpal floor. The cavity then was evaluated for any residual softened dentin tissue. The procedure was repeated as needed to eliminate all infected dentinal caries. The healthy dentin was confirmed when the gel no longer changed color and turned turbid followed by visual-tactile clinical criteria (Ericson et al. 1999).

#### Experiment 2 (caries removal utilizing Carie-Care™)

The carious cavity was treated with Carie-Care™ which was applied directly on the cavity via plastic disposable tips. Following the instructions of the manufacturer, the Carie-Care™ gel was brought to room temperature by taking it out from the ice cooler 15–20 min prior to the clinical session. The gel was left in the carious lesion for 2 min. During that time, the appearance of the gel should be changed from translucent into cloudy with the formation of oxygen bubbles. Then a moistened cotton pellet was used to remove the gel. A dry, sterile cotton pellet and a gentle air blow were used to dry the cavity after it had been washed with water. The softened caries were excavated by a dental excavator. The procedure could be repeated as required till the gel no longer changed color followed by visual-tactile clinical criteria (Ericson et al. 1999).

#### The control group (caries removal utilizing conventional method)

Sterilized low-speed tungsten carbide round burs on a slow-speed handpiece with water cooling (Max 30.000 rpm) and high-speed tungsten carbide round burs on a high-speed handpiece with water cooling (Max 300.000 rpm) were used for removing caries and undermined enamel in both experiments. After complete caries removal, the cavity was confirmed to be caries-free according to visual and tactile clinical criteria using a dental explorer (Ericson et al. 1999).

Dentin was considered to be caries free with the CMCR methods when the gel no longer changed color and turned turbid followed by utilizing visual (lack of discolored dentin) and tactile (easy movement of the probe with no feeling of tug back or catch) clinical criteria by a dental explorer (Ericson et al. 1999). Following complete caries removal, teeth were restored with glass-ionomer restoration (GC Fuji IX GP^®^) based on the manufacturer’s instructions then transferred to the post-graduate pediatric dental clinics for the final restorations.

### Assessment procedures

The caries removal time was measured in minutes by a stopwatch from the start of caries removal until a caries-free cavity was ascertained using the visual-tactile clinical criteria in addition to the color-changing criteria for CMCR agents. Pain perception throughout caries removal was assessed in all groups using the Arabic version of the “Wong-Baker FACES^®^ Pain Rating Scale” (Wong and Baker 1988). The scale comprises six faces labelled 0, 2, 4, 6, 8, and 10, representing a spectrum of pain expression from 0, denoting no pain, escalating to 10, signifying the worst pain imaginable. Before starting the dental procedure, the pediatric dentist explained this scale to each patient by pointing to each face and describing the degree of pain intensity that face was showing. Immediately following the dental procedure, the child pointed to the facial image that reflected his/her level of pain and how he was feeling during caries removal following the question, "How did you feel during treatment?" then the appropriate number was recorded.

### Statistical analysis

The statistical evaluation was done utilizing Statistical Package for Social Sciences (SPSS, V.20, IBM; Armonk, NY, USA). The working time data was normally distributed. Therefore, the working time between groups was compared within each experiment using the paired sample t-test. An independent sample t-test was used to compare the working times of the CMCR agents between experiment 1 and 2. The Wong-Baker FACES^®^ Pain Rating Scale pain perception data ranging from 0 to 10 did not follow a normal distribution. The pain perception between groups was evaluated within each experiment using the Wilcoxon Signed Rank test. A Mann–Whitney *U* test was utilized to compare the pain perception of the CMCR agents between experiment 1 and 2.

## Results

**Fig. 1:**
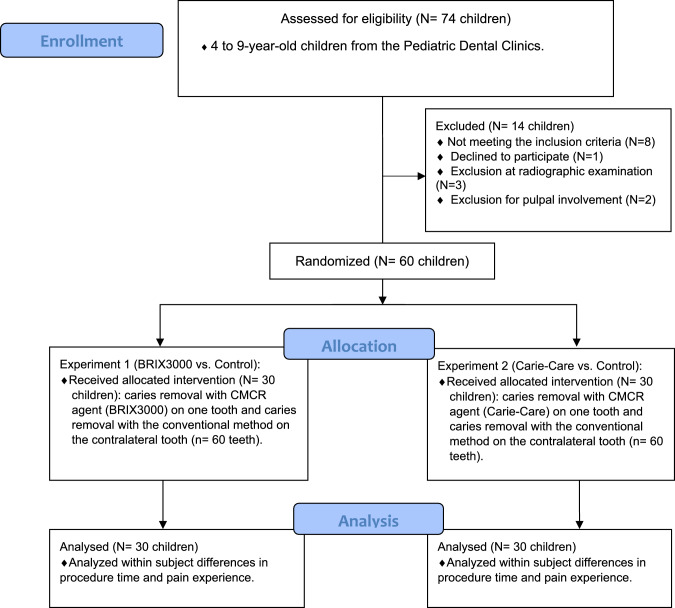
Flow diagram of the study

This study consisted of healthy and cooperative children between the ages of 4–9 years with bilateral cavitated occlusal caries in primary molars. A total of 120 primary molars were included (60 teeth for experiment 1 and 60 teeth for experiment 2). The flow diagram of the study, according to the "Consolidated Standards of Reporting Trials" (CONSORT, 2010) statement, shows the total number of subjects and teeth in the study (Fig. 1).

Among the 60 selected children, the range of age was from 4 to 9 years, and the mean age was 6.00 ± 1.62 years. The study sample consisted of 45.00% boys and 55.00% girls. Table 1 demonstrates the frequency and percentage distribution of the subjects according to age and sex.

**Table 1:** Distribution of the included subjects according to age and sex

Parameter	Total *N = *60 subjects	Experiment 1 *N = *30 subjects	Experiment 2 *N = *30 subjects
Age (years)	*N* (%)		
4–6	33 (55.00)	18 (60.00)	15 (50.00)
7–9	27 (45.00)	12 (40.00)	15 (50.00)
Sex	*N* (%)		
Male	27 (45.00)	13 (43.33)	14 (46.67)
Female	33 (55.00)	17 (56.67)	16 (53.33)

*N* number of subjects, *Experiment 1* BRIX3000^®^ vs. conventional, Experiment 2 Carie-Care™ Vs. conventional

A total of 120 teeth were treated in both groups. In experiment 1, the mandibular teeth were more commonly treated in BRIX3000^®^ group (53.33%) and the conventional group (60.00%). In experiment 2, the maxillary teeth were more commonly treated teeth in Carie-Care™ group (53.33%) and the mandibular teeth were more commonly treated teeth in the conventional group (60.00%). No statistically significant difference in distribution between the maxillary and mandibular arches, as well as between the first and second primary molars, was detected among groups. Table 2 presents the frequency and percentage distribution of teeth by arch and tooth type among the two groups.

**Table 2:** Frequency (*n*) and percentage (%) distribution of teeth by arch and tooth type among the two experiments

	Experiment 1 *n* (%)		Experiment 2 *n* (%)	
	BRIX3000® *n = *30 teeth	Conventional *n = *30 teeth	Carie-Care™ *n = *30 teeth	Conventional *n = *30 teeth
Arch				
Maxillary	14 (46.67)	12 (40.00)	16 (53.33)	12 (40.00)
Mandibular	16 (53.33)	18 (60.00)	14 (46.67)	18 (60.00)
*P* value^a^	0.602		0.301	
Tooth				
1st primary molar	14 (46.67)	14 (46.67)	14 (46.67)	15 (50.00)
2nd primary molar	16 (53.33)	16 (53.33)	16 (53.33)	15 (50.00)
*P *value^a^	1.00		0.796	

^a^Chi-square test

**Fig. 2:**
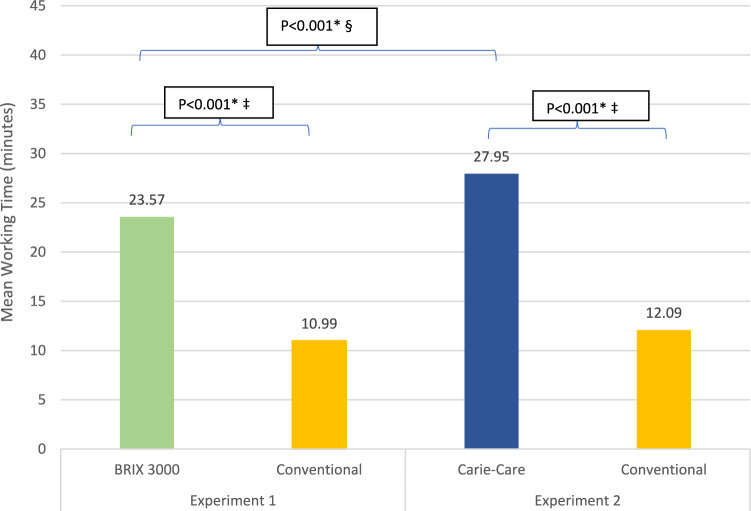
Comparison of the mean working time among the two experiments. *Statistically significant (*P < *0.05), ‡ Paired sample t-test, § Independent sample t-test

Caries removal time data was found to be normally distributed. Therefore, in experiment 1, the mean duration of treatment needed for caries removal in the CMCR method BRIX3000^®^ was 23.57 ± 4.45 min while the mean duration of treatment in conventionally treated teeth was 10.99 ± 2.97 min with a statistically significant difference (*P < *0.001) utilizing the paired t-test. In experiment 2, similarly, the conventional method (12.09 ± 2.67 min) was more time efficient than the CMCR method Carie-Care™ (27.95 ± 4.27 min) in caries removal with statistically significant difference (*P < *0.001). Additionally, using an independent group design, the CMCR method BRIX3000^®^ in experiment 1 was more time efficient than the CMCR method Carie-Care™ in experiment 2 with a statistically significant difference (*P < *0.001) utilizing independent sample t-test. The mean working time for teeth treated with the CMCR method BRIX3000^®^, the CMCR method Carie-Care™, and the conventional method is summarized in Fig. 2.

**Fig. 3:**
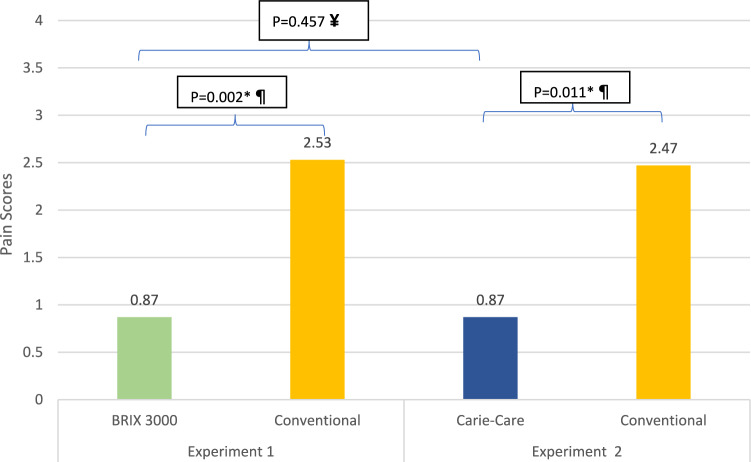
Comparison of the mean pain scores according to the “Wong-Baker FACES^®^ Pain Rating Scale” during caries removal among the two experiments. *Statistically significant (*P < *0.05), ¶ Wilcoxon signed rank test, **¥**Mann–Whitney U test

Pain perception data was found to be non-normally distributed. In experiment 1, the mean pain score during caries removal in teeth treated with the CMCR method BRIX3000^®^ was less painful with a mean score of 0.87 ± 1.25 while the mean pain score during caries removal in teeth treated conventionally was 2.53 ± 2.92 with statistically significant difference (*P* = 0.002) using Wilcoxon signed-rank test. In experiment 2, CMCR method Carie-Care™ during caries removal was also less painful (0.87 ± 2.01) than the conventional method (2.47 ± 2.71) with statistically significant difference (*P* = 0.011) using Wilcoxon signed-rank test. Additionally, using an independent group design, the CMCR methods with either BRIX3000^®^ agent or Carie-Care™ showed no statistically significant difference in pain perception (*P* = 0.457) using Mann Whitney U test. The “Wong-Baker FACES^®^ Pain Rating Scale” score for teeth treated with the CMCR method BRIX3000^®^, the CMCR method Carie-Care™, and the conventional method is summarized in Fig. 3.

## Discussion

The findings of the present study demonstrated that the utilisation of the CMCR technique with BRIX3000^®^ or Carie-Care™ resulted in significantly reduced pain during caries removal in comparison to the conventional method. Further, caries removal using the CMCR method with BRIX3000^®^ or Carie-Care™ took much longer than with the conventional method. The null hypothesis was rejected. The present study utilised an experimental, randomised, controlled clinical trial with two distinct experiments conducted using a split-mouth design. In each experiment, teeth were randomly assigned to one of two study groups and then evaluated for efficiency and pain perception during caries removal. The split-mouth design enhances the strength of the study by minimising variations among subjects and eliminating the operator's preference for a specific site, thus minimizing the risk of bias (Ofori-Asenso and Agyeman 2015).

The results of this study corroborate those of other studies. In a split-mouth randomized controlled clinical trial, BRIX3000^®^ was discovered to be a successful alternative approach for caries eradication that appears to be more patient-friendly and conservative than the conventional method of using low-speed air-rotors with ceramic burs in the permanent molars of children aged 8–12 years, despite the slightly longer procedure (Ismail and Al Haidar 2019). The Carie-Care™ procedure took longer than the conventional method in the first permanent molar in another split-mouth clinical trial involving healthy children aged 6–9 years old, but the children reported less pain on the Wong-Baker FACES^®^ Pain Rating Scale (Nalawade et al. 2019). In the randomized controlled clinical trial conducted by Sontakke et al. (2019), it was found that Carie-Care™, although more time-consuming, was significantly more favorable than the conventional method for children between the ages of 12 and 15 years (Sontakke et al. 2019). A different randomised controlled clinical trial found that conventional treatment took less time to remove caries compared to Brix3000^®^ and NaOCl gel in children aged 6–9 years with proximal caries in primary maxillary molars. The study also found that CMCR agents effectively removed carious dentin from primary teeth without adversely affecting children's cooperation (Alkhouli et al. 2020). The findings of our study may be ascribed to numerous factors. The CMCR approach removes only carious dentin, whereas the conventional method removes both sound and carious dentin. This may account for the lack of pain or very little pain felt when using the CMCR method to remove caries. Moreover, rapid excavation of cavities often results in the excessive removal of healthy tooth material, which can lead to pain due to the proximity of the bur to the pulpal tissues (Pathivada et al. 2016). Furthermore, the absence of vibration or heat production when employing the CMCR method led to minimal or no widening of dentinal tubules, which can be attributed to a limited pulpal reaction (Bohari et al. 2012).

However, the study results contradict a previous study that concluded there was no statistically significant distinction in pain perception and patient behavior between the conventional method and Papacárie^®^ (Matsumoto et al. 2013). Meanwhile, Almaz et al. (2016) employed a questionnaire to compare the pain of Papacárie^®^ and the conventional method. Their findings revealed no statistically significant difference in pain between the two methods. However, it is important to note that the pain evaluation method used in the study was subjective (Almaz et al. 2016). In their study, Kotb et al. (2009) discovered that when considering the time required for LA, Papacárie^®^ CMCR actually took significantly less time compared to the conventional method (Kotb et al. 2009).

In both experiments of the present study, LA was administered. In standard dental procedures, the removal of caries using conventional devices such as high- and low-speed handpieces and burs necessitates the use of LA due to the anticipated dental anxiety and pain caused by the removal of both infected and affected dentin (Venkataraghavan et al. 2013). The handpieces' accompanying noise and vibration also contribute to the dental anxiety and pain experienced with the conventional technique. Furthermore, the heat produced during the cutting procedure can have a detrimental impact on the pulp, resulting in pulpal pain (Banerjee et al. 2000; Maragakis et al. 2001). Consequently, it was ethically necessary to administer LA in the conventional groups. The primary aim of this study was to examine the pain perception experienced during caries removal using the CMCR agents BRIX3000^®^ or Carie-Care™ compared to the conventional method. It is not possible to achieve this objective if LA is only given during the conventional procedure. In such cases, patients experience significant pain due to the injection, which can potentially distort the results of pain perception. The present study examined the pain response specifically related to the overall pain experience during the clinical procedure. This pain experience may be caused by the discomfort resulting from factors such as noise, vibration, heat, and pressure during excavation, as well as the duration of treatment. It is important to note that this pain experience is separate from the pain caused by the injection. Consequently, LA was administered to both the experimental and control groups to ensure consistency, despite the manufacturers of the CMCR agents, such as BRIX3000^®^ and Carie-Care™, advising against the use of LA. This was based on a study that utilized LA for both groups to evaluate the effectiveness of caries removal, both clinically and microbiologically, using a polymer bur and Carie-Care™ on the primary second molars of 25 children aged 5–9 years (Aswathi et al. 2017).

In the present study, certain limitations can be discerned. A limitation of this research may be the absence of standardization in the size of the lesions. While the CMCR method generally adheres to the principles of MID, there were a few instances where it was necessary to utilize high-speed handpieces to expand the cavity and allow for proper access to the CMCR agents. Since these occurrences were extremely uncommon, no statistical analysis was done to determine whether they influenced the outcomes. A further limitation is that the current study was unable to implement blinding due to the inherent characteristics of the clinical procedures. The CMCR agents merit additional scrutiny, particularly within a clinical context. Future research should focus on enzyme-based products, specifically BRIX3000^®^, due to their growing popularity. Simultaneous evaluation of CMCR techniques with the conventional method and other MID strategies is necessary for both primary and permanent teeth. Additional clinical trials are necessary to evaluate the financial implications and lifespan of restorations.

## Conclusions

Within the limitations of the present randomized controlled trial, it has been shown that the CMCR technique of caries removal using either “BRIX3000^®^” or “Carie-Care^TM^” resulted to less pain compared to the conventional method of using handpieces. The CMCR methods required longer treatment time for caries removal in comparison to the conventional method. This technique appears having the potential to be an alternative treatment option to the conventional method, particularly for those anxious children requiring dental treatment.

## Data Availability

The datasets used and analyzed during the current study are available from the corresponding author upon reasonable request.
